# Equity and budget challenges associated with the employment of anti-amyloid monoclonal antibodies for early Alzheimer’s Disease in Italy: A scenario analysis

**DOI:** 10.1016/j.jarlif.2026.100076

**Published:** 2026-06-04

**Authors:** Maurizio Giorelli, Pasquale Di Fazio, Tiziana Dimatteo

**Affiliations:** a Operative Unit of Neurology, “Dimiccoli” General Hospital, Barletta, ASL BT, Italy; b Operative Unit of Nuclear Medicine, “Dimiccoli” General Hospital, Barletta, ASL BT, Italy; c General Director, ASL BT, Italy

**Keywords:** Alzheimer's disease, Anti-amyloid monoclonal antibodies, Health economics, Implementation science, Health equity, Italy, Healthcare policy

## Abstract

**Background:**

Anti-amyloid monoclonal antibodies (lecanemab and donanemab) represent the first disease-modifying therapies for early symptomatic Alzheimer's disease (AD)- Italy's National Health Service (SSN) must determine whether to include these therapies within Essential Levels of Care (LEA), requiring reimbursement across all regions.

**Objective:**

To conduct a three-year budget impact analysis and implementation feasibility assessment for anti-amyloid therapies in Italy. The analysis was scenario-based rather than empirical.

**Methods:**

Deterministic budget modeling, combined with Proctor's 8-Domain Implementation Outcomes Framework and the Consolidated Framework for Implementation Research (CFIR), was employed. Four implementation scenarios were modeled based on cumulative patient uptake rates. Cost analysis incorporated diagnostic pathways, drug acquisition, clinical surveillance, and management of amyloid-related imaging abnormalities (ARIA). Infrastructure capacity was assessed through national survey data of Centers for Cognitive Disorders and Dementia (CCDDs).

**Results:**

An estimated 42,000 patients in Italy are potentially eligible for anti-amyloid therapy. The moderate scenario (5–10–15% uptake over three years, reaching 14,100 cumulative patients) represents the optimal policy target, with estimated three-year total costs of €557.3 million (0.4% of the annual SSN budget), penetration of 33.6% of eligible patients, and a feasibility score of 1.5, underscoring a high implementation success probability. The moderate scenario remains financially sustainable but requires targeted infrastructure investment in the Central and Southern regions.

**Conclusions:**

Anti-amyloid therapy implementation is feasible within Italian healthcare system constraints under a moderate uptake scenario. However, achievement of equitable access requires substantial capacity building in underserved regions, clarified reimbursement policies, integrated diagnostic networks, and robust workforce planning before full LEA inclusion is recommended.

## 1 Introduction

Alzheimer's disease (AD) represents a significant global neurodegenerative challenge affecting approximately 49 million individuals aged 65 years and older, with prevalence expected to increase substantially in the coming decades [1]. The advent of anti-amyloid monoclonal antibodies (mAbs), including lecanemab and donanemab, marks a paradigm shift in AD management, moving from symptomatic treatments to disease-modifying approaches that target underlying amyloid pathology [2]. These represent the first treatments to demonstrate clinically meaningful cognitive benefits in early symptomatic stages (mild cognitive impairment and mild dementia). In the CLARITY AD trial, lecanemab reduced clinical decline by approximately 27% over 18 months compared with placebo [3]. Similarly, the TRAILBLAZER-ALZ 2 trial reported clinically meaningful slowing of disease progression with donanemab [4].

Italy's National Health Service (Servizio Sanitario Nazionale, SSN) provides universal healthcare through regionalized governance, with each region responsible for implementing essential care standards defined by the central government. Livelli Essenziali di Assistenza (LEA), updated for 2024–2025, specify which treatments and services must be reimbursed universally across all 20 regions. Inclusion within LEA represents the highest-level policy decision, mandating equitable access to treatment nationwide.

Decisions to include anti-amyloid mAbs within LEA will determine whether these therapies are universally reimbursed across the SSN or remain restricted to private or regional pilot programs. Italy currently serves approximately 413,000 patients with Alzheimer's disease at direct annual costs exceeding €1.8 billion [5]. Regional dementia care pathways remain fragmented, with diagnostic capacity concentrated in Northern Italy, creating substantial geographic disparities in access to specialist evaluation and emerging treatments. The critical policy question is: Can Italy provide anti-amyloid monoclonal antibodies within the SSN's LEA framework in a way that is economically sustainable and equitable across all regions and socioeconomic groups?

Within this critical context, the fundamental policy challenge for Italian healthcare planners is to ensure sustainability and equity in the implementation of anti-amyloid therapies (ATTs) across the country, while overcoming substantial limitations and regional differences stemming from varying local and regional healthcare workforce capacities [6,7].

Patient eligibility requires confirmation of biomarkers through cerebrospinal fluid analysis or positron emission tomography. Intensive monitoring protocols are required, including magnetic resonance imaging surveillance for amyloid-related imaging abnormalities (ARIA), which occur in 17–21% of treated patients with approximately 2–5% requiring clinical intervention at costs ranging from €3000–€8000 per symptomatic event [8].

The substantial direct costs—ranging from €25,000 to €30,000 annually for drug acquisition alone, coupled with diagnostic, monitoring, and infrastructure costs—raise fundamental questions about equitable access, healthcare system sustainability, and appropriate resource allocation in countries with constrained healthcare budgets [5].

This is a policy-oriented, structured scenario analysis, not an empirical impact study. We employ deterministic budget modeling combined with Proctor's Domain Implementation Outcomes Framework and Consolidated Framework for Implementation Research (CFIR) to simulate budget impacts under realistic uptake scenarios, and to identify barriers to fair access [9,10].

This scenario-based policy analysis is designed to inform Italy's 2026–2027 deliberations on the inclusion of anti-amyloid therapies within the Essential Levels of Care (LEA) framework. It provides a structured assessment of budgetary implications, infrastructure readiness, and equity considerations, drawing on available Italian epidemiological data, published trial results, and established implementation frameworks. The analysis identifies policy prerequisites and feasibility constraints over the next 3 years, rather than predicting specific outcomes or prescribing definitive implementation pathways.

## 2 Methods

### 2.1 Population eligibility

We derived estimates of the eligible population from Italian epidemiological data on mild cognitive impairment (MCI) prevalence and real-world trial eligibility rates. In 2024, the National Institute for Statistics (ISTAT) measured a total of 58,934,000 inhabitants in Italy, 25% (14,733,000) of whom were aged 65 years and over. Previous studies reported a prevalence of MCI of 7.7% in individuals older than 65 years in Italy, which would mean a total of 1134,441 MCI patients [11]. Studies on the prevalence of MCI in people from Southern Italy aging over 60 years reported a similar estimate, with amnestic MCI weighing for 7.4% of the interested population (95% CI: 5.8–9.4%). When considering reported estimates for AD burden worldwide, prodromal AD is thought to affect 41% of MCI individuals in Italy, namely 465,121 [6,12].

A tertiary Italian center assessed 408 patients with MCI or mild AD against donanemab trial eligibility criteria, finding that 41% were amyloid-positive but only 10.05% met full treatment eligibility due to biomarker status, comorbidities, and neuroimaging findings [12]. Recent studies on the frequency of APOE ε4/4 carriers in Italy found a substantially lower frequency than in Northern European countries: Italians from the Central and Southern regions had an 8.3% frequency of APOE ε4/4 status, while people from Sardinia had a 5.2% frequency [13]. Considering a mean of 8% frequency in Italy, we may estimate that approximately 9% of the whole prodromal AD would be truly eligible patients, namely they would range around 42,000 patients. These represent the 0.3% of the population aged 65 and older. For estimates in this study, we conservatively use 42,000 CE patients potentially eligible for ATT in Italy. Estimates of eligibility are synthetized in Table 1.

**Table 1:** Patient eligibility calculation.

Population Metric	Number	Derivation
Total Italian Population (2024)	58,934,000	ISTAT data
Population aged ≥65 years	14,733,000	25% of total population
MCI Prevalence (≥65 years)	1,134,441	7.7% of ≥65 population
Prodromal AD (41% of MCI)	465,121	Estimate from AD burden data
Full Treatment Eligibility (10.05% of assessed)	42,000	9% adjusted for APOE ε4/4 frequency and comorbidities
Eligible Population as % of ≥65 years	0.3%	42,000 / 14,733,000

### 2.2 Time horizons and healthcare system readiness

We modeled a 3-year budget envelope (2026–2028) to assess near-term implementation feasibility and infrastructure readiness. This 3-year horizon reflects the immediate policy window for national reimbursement decisions and regional capacity planning.

A survey of Italian Centers for Cognitive Disorders and Dementia (CCDDs) identified 534 centers nationwide. Of these, 223 CCDDs (41.8%) are in Northern regions, 105 (19.7%) in Central regions, and 206 (38.5%) in Southern regions and Islands [14]. Of all CCDDs, 450 provided complete information about staff and services. Forty-seven (47) of them met all requirements for effective anti-amyloid therapy prescription and administration, with 33 located in Northern Italy, thereby creating geographic inequities in which patients' residence determines access to diagnostic evaluation.

CCDDs capable of anti-amyloid therapy (ATT) prescription differed from those not qualifying in "better organizational aspects and activity profile," including number of opening hours, presence of psychologists, and digital information systems; better "services provided to patients" including availability of blood tests, Magnetic Resonance Imaging, Computerized Tomography, PET FDG, PET amyloid, and biomarker assessments on cerebrospinal fluid (CSF) and plasma [6]. A critical attribute differentiating CCDDs was the significant patient-to-center ratio in Southern Italy. For example, CCDDs in Umbria (n = 2) managed 7539 MCI patients, compared with 79,104 in Campania (n = 1), indicating extreme capacity constraints in high-population-density Southern regions [14].

### 2.3 Budget impact analysis

In our budget analysis, we employed 2024–2025 Italian National Health Service Essential Levels of Care (LEA) tariffs and a bottom-up costing approach.

Annual per-patient costs comprised: (a) diagnostic pathway = €1692 (estimated); (b) drug acquisition (€27,500 annually); (c) clinical surveillance, including monthly specialist consultations and six annual brain MRI scans for ARIA monitoring (€ 2000). ARIA management costs for symptomatic cases—hospitalization, neurological assessment, immunosuppression, and repeat imaging—are estimated at €3000–€8000 per symptomatic event [8], occurring in 2–5% of treated patients annually.

Using these tariffs, we modeled four implementation scenarios based on cumulative patient treatment over three years. Of the 42,000 eligible patients, we hypothesized: (1) Minimal scenario with 1% annual uptake (1410 cumulative patients by year 3); (2) Conservative scenario with 2–4–6% uptake (5640 cumulative); (3) Moderate scenario with 5–10–15% uptake (14,100 cumulative); and (4) Optimistic scenario with 10–20–30% annual uptake (28,200 cumulative). Feasibility was ranked based on healthcare system readiness, diagnostic capacity, workforce availability, and international benchmarks [14].

### 2.4 Probabilistic sensitivity analysis

A probabilistic sensitivity analysis was conducted to explore joint uncertainty in budget impact estimates. Key model parameters were assigned probability distributions according to parameter type. Cost parameters, including drug acquisition, diagnostic assessment, monitoring, and ARIA management costs, were modelled using gamma distributions. Eligible population size and scenario-specific treatment uptake were modelled using truncated normal distributions.

For each implementation scenario, 10,000 Monte Carlo simulations were performed over the 3-year analytic horizon. In each iteration, all uncertain parameters were sampled simultaneously, and the total cumulative expenditure was recalculated. Results were reported as mean 3-year costs with 95% uncertainty intervals. We also estimated the probability that each scenario remained below 1.0%, of annual SSN expenditure.

This analysis was used to assess whether the relative affordability of the modeled scenarios was robust to simultaneous uncertainty in epidemiological, pricing, diagnostic, monitoring, and ARIA-related assumptions.

### 2.5 Implementation framework assessment: proctor and CFIR

Using Proctor's 8 Domain Implementation Outcomes Framework [9], we assessed four core implementation criteria because their evaluation is more closely linked to empirical data, such as known expenses or organizational facilities. Domains considered were feasibility, cost efficiency, penetration, and sustainability. Domains were operationalized on a 0–2 scale. Scoring criteria were: 0 = target not met; 1 = partially achieved; 2 = fully addressed.

Specific operationalization:

• Feasibility: scored 0 if current infrastructure cannot support the scenario without major reconstruction, 1 if modest capital investment could enable implementation, 2 if existing systems and workforce could accommodate the scenario.
• Cost efficiency: scored 2 if the probability of annual total cost 〈1% of the SSN annual budget was 〉 90% (approximately € 140 billion), 1 if probability ranged 70–90%, 0 if < 70%.
• Penetration: scored based on percentage of eligible population reached; 0 if <10%, 1 if 10–40%, 2 if >40%.
• Sustainability: scored 0 if scenario dependent on temporary funding or external grants, 1 if partial integration into standard care, 2 if sustainable within existing budget frameworks with modest reallocation.

Domain scores were averaged to generate success probabilities: mean score >1.5 = high; 1.5 = moderate; <1.5 = low. These represent expert-judgment assessments of implementation readiness based on structural feasibility, not statistically estimated probabilities from empirical data.

We applied the Consolidated Framework for Implementation Research (CFIR) [10] as a structured lens to categorize equity barriers across five domains: (1) Outer Setting (healthcare context, geography, policy environment), (2) Inner Setting (organizational capacity, resources, existing workflows), (3) Individuals (patient and provider characteristics), (4) Innovation (intervention characteristics and complexity), and (5) Implementation Process (rollout strategy, fidelity monitoring, quality assurance). This stratification identified which barriers were systemic, organizational, or individual, informing targeted policy solutions.

## 3 Results

### 3.1 Budget impact analysis by scenario

Three-year cumulative budget projections across all four implementation scenarios are presented in Table 2.

**Table 2:** Budget impact analysis by scenario.

Implementation Scenario	Annual Uptake %	Cumulative Patients (Year 3)	Total 3-Year Mean Cost (€ Million)	% of Annual SSN Budget	95% uncertainty interval	Probability < 1% SSN	Population Penetration %
Minimal	1%	1410	€79.4	0.06%	€ 49.2–118.6	99.9%	3.4%
Conservative	2–4–6%	5640	€219.7	0.16%	€ 136.8–331,5	99.8%	13.4%
Moderate	5–10–15%	14,100	€557.3	0.4%	€ 341.7- 835.9	94.6%	33.6%
Optimistic	10–20–30%	28,200	€1112	0.9%	€ 678.2–1684	61.8%	67.1%

The Minimal scenario (1% annual uptake) yields 1410 cumulative treated patients by year 3, with a mean three-year cost of €79.4 million, representing 0.06% of the annual SSN budget and reaching only 3.4% of the eligible population. While financially negligible, this scenario fails to address patient needs and constitutes suboptimal resource allocation despite demonstrated therapeutic benefit.

The Conservative scenario (2–4–6% annual uptake) treats 5640 cumulative patients over three years with a mean total cost of €219.7 million (0.16% of SSN budget), achieving 13.4% population penetration. This scenario reflects cautious uptake aligned with international conservative benchmarks for novel disease-modifying therapies in early adoption phases.

The Moderate scenario (5–10–15% annual uptake) represents the evidence-based optimal policy target, treating 14,100 cumulative patients with three-year costs of €557.3 million (0.4% of annual budget). This scenario achieves 33.6% penetration of the eligible population—a level consistent with second-phase adoption of effective therapies once initial infrastructure and provider-familiarity barriers are surmounted. Critically, annual costs remain <€200 million in all three years, well within feasibility parameters.

The Optimistic scenario (10–20–30% annual uptake) treats 28,200 patients with three-year costs of €1.112 billion (0.9% of SSN annual budget), achieving 67.1% penetration. While this demonstrates maximal treatment benefit at the population level, it approaches the 1.5% three-year cost threshold and would require substantial simultaneous infrastructure expansion across multiple regions, a scenario we assess as operationally unlikely within realistic workforce-planning horizons.

Annual cost trajectories across scenarios show the expected escalation over the study period as the cumulative number of treated patients increases. In the moderate scenario specifically, year 1 costs total €121.4 million, year 2 costs €190.2 million, and year 3 costs €233.9 million, reflecting the accumulation of both new diagnostic evaluations and ongoing surveillance of previously treated patients. ARIA management costs in the moderate scenario total €4.8 million over three years (0.88% of total intervention costs), distributed proportionally across treated patients.

Diagnostic pathway costs constitute a critical but often underestimated component of implementation. In the moderate scenario, three-year diagnostic costs total €66.8 million (12.3% of total intervention costs). This emphasizes the substantial infrastructure investment required beyond drug acquisition alone—a factor that disproportionately impacts regions lacking integrated diagnostic networks [5,6].

Probabilistic sensitivity analysis confirmed that treated population size and anti-amyloid acquisition cost were the dominant drivers of financial uncertainty. Minimal and conservative implementation scenarios remained below 1% of annual SSN expenditure in nearly all simulations. The moderate, phased implementation scenario remained below this threshold in most iterations, whereas the optimistic rollout scenario exhibited substantially greater financial uncertainty and a significantly higher probability of exceeding predefined affordability thresholds.

### 3.2 Proctor framework scoring and implementation success probabilities

Implementation success probabilities derived from Proctor domain scoring are presented in Table 3.

**Table 3:** Proctor framework implementation scoring & success probabilities.

Scenario	Feasibility Score	Cost Efficiency Score	Penetration Score	Sustainability Score	Mean Score	Implementation Success Probability
Minimal	2	2	0	2	1.5	moderate
Conservative	1.5	2	1	1.5	1.5	moderate
Moderate	1.5	2	2	1.5	1.75	high
Optimistic	1	0	2	1	1	low

The Minimal scenario achieves a mean Proctor score of 1.75 (Feasibility: 2, Cost Efficiency: 2, Penetration: 0, Sustainability: 2), corresponding to moderate implementation success probability. The zero-penetration score reflects failure to address the policy objective of equitable patient access.

The Conservative scenario yields a mean score of 1.5 (Feasibility: 1.5, Cost Efficiency: 2, Penetration: 1, Sustainability: 1.5), corresponding to moderate success probability. This represents the threshold at which implementation feasibility is assured and modest patient benefit is addressed.

The Moderate scenario achieves a mean score of 1.75 (Feasibility: 1.5, Cost Efficiency: 2, Penetration: 2, Sustainability: 1.5), underscoring a high success probability threshold while uniquely achieving full penetration scoring (>40% of the eligible population reached).

The Optimistic scenario receives a mean score of 1 (Feasibility: 1, Cost Efficiency: 1, Penetration: 2, Sustainability: 1), corresponding to 70–85% success probability due to a genuine risk of implementation failure if external conditions (economic recession, workforce constraints, policy changes) materialize.

## 4 CFIR equity barriers by implementation domain

Application of the Consolidated Framework for Implementation Research (CFIR) identified significant equity barriers clustered across five domains (Table 4):

**Table 4:** CFIR equity barriers assessment - five-domain framework.

CFIR Domain	Core Equity Barriers	Primary Affected Population
Outer Setting—Healthcare Context & Geography	>70% treatment centers in Northern Italy; Southern patients travel 500+ km; €410–€1700 annual transport costs; absent standardized biomarker cutoffs and national policy commitment	Elderly patients with mobility constraints, Southern Italy residents, and low-income populations
Inner Setting—Organizational Capacity	personnel shortage; neuroradiology expertise concentrated in academic centers; workflows designed for diagnosis, not longitudinal management; cumulative visit burdens	Vulnerable elderly; lower-income, lower-education, reduced-mobility, caregiving-constrained populations
Individuals—Patient & Provider Characteristics	Socioeconomic status predicts access; health literacy and provider knowledge gaps; marginalized populations experience diagnostic delays; elderly immigrants face interpreter unavailability and discrimination	Marginalized populations; elderly immigrants; patients with limited health literacy and restricted healthcare access
Innovation—Intervention Characteristics	Anti-amyloid therapy exceeds primary care capacity; requires biomarker confirmation, specialist visits, MRI, PET/CSF, infusion centers, ARIA management; incompatible with cognitively impaired or geographically isolated patients	Patients with cognitive impairment; geographically isolated populations; limited digital health literacy
Implementation Process—Rollout Strategy	"First-come, first-served" access favors academic centers; absent national dementia registry; no standardized ARIA protocols or equity audits before expansion	Patients distant from academic centers; unregistered or late-diagnosed patients; underserved populations

### 4.1 Outer setting—healthcare context & geography

Northern Italy concentrates >70% of treatment-capable centers despite having only 41.8% of all diagnostic centers. This creates stark disparities: Campania's 79,104 MCI patients rely on a single center, while Umbria's 7539 patients have two centers. Southern patients face 500+ km travel for biomarker testing, incurring €410–€1700 annually in transportation, lodging, and lost wages—prohibitive for elderly populations with mobility constraints. Lack of standardized biomarker cutoffs across regions and the absence of national policy commitment to anti-amyloid therapy further entrench geographic inequities, requiring systemic reorganization rather than individual solutions.

### 4.2 Inner setting—organizational capacity

The system cannot support intensive therapy. Across 534 diagnostic centers, 300–390 personnel (specialists, neuroradiologists, nurses) will be needed within 3–5 years. Neuroradiology expertise concentrates in academic centers, creating surveillance bottlenecks. Current workflows serve diagnosis, not longitudinal drug management and ARIA monitoring. Vulnerable elderly, those with lower income, limited education, reduced driving ability, or caregiving constraints, face cumulative burdens across repeated visits for baseline evaluation, biomarker confirmation, infusions (monthly/quarterly), and MRI surveillance.

### 4.3 Individuals—patient & provider characteristics

Socioeconomic status directly predicts access. Health literacy disparities affect informed consent; provider knowledge varies sharply between academic and peripheral settings. Evidence confirms that education and employment influence biomarker testing uptake and specialist access. Marginalized populations, lower education, limited health literacy, restricted healthcare access, experience diagnostic delays and miss optimal intervention windows. Elderly immigrants face compounded barriers: interpreter unavailability, culturally inappropriate materials, and potential provider discrimination.

### 4.4 Innovation—intervention characteristics

Anti-amyloid therapy exceeds primary care capacity. Requirements include biomarker confirmation, three annual specialist visits, regular MRI, specialized imaging (PET/CSF), infusion centers, and ARIA management with potential hospitalization—incompatible with patients facing cognitive impairment, geographic barriers, or limited digital health literacy.

### 4.5 Implementation process—rollout strategy

Without coordinated sequencing, "first-come, first-served" access will favor patients near academic centers. Absent national dementia registry prevents prospective equity monitoring. Without standardized ARIA protocols, caregiver support, and equity audits before expansion, rapid rollout will concentrate treatment in Northern Italy, widening existing disparities.

## 5 Discussion

### 5.1 Interpretation of budget impact findings

This analysis demonstrates that anti-amyloid therapy implementation in Italy is financially feasible under realistic uptake scenarios. The moderate scenario, treating 14,100 patients over three years at a cumulative cost of €557.6 million, consumes 0.4% of the SSN's annual budget, a level consistent with the health system's capacity to absorb new interventions targeting serious diseases with demonstrated therapeutic benefit [10].

However, financial feasibility alone is insufficient justification for implementation. The moderate scenario is recommended because it optimally balances four policy objectives: (1) financial sustainability within SSN constraints, (2) meaningful reduction of cognitive decline for one-third of eligible patients, (3) high achievement of implementation success probability based on realistic infrastructure assessment, and (4) provision of a foundation upon which future expansion can build once workforce and diagnostic capacity are strengthened.

Critical to this interpretation is the recognition that diagnostic and monitoring costs, €66.8 million in the moderate scenario, represent substantial infrastructure investment beyond drug acquisition. Many healthcare systems underestimate these costs when evaluating disease-modifying therapies, creating post-implementation budget surprises and care delays. Italy's integrated SSN structure provides opportunity to establish coordinated diagnostic networks that could achieve efficiencies unavailable in fragmented healthcare systems; however, this requires explicit planning beyond this analysis.

### 5.2 Implementation feasibility and regional equity implications

The analysis reveals that the feasibility of anti-amyloid therapy varies by region. Northern Italy possesses adequate, qualified infrastructure (33 of 47 centers, 70.2%) to implement the moderate scenario without major reconstruction; infrastructure constraints are real but manageable through modest additions to existing service capacity. The Northern regions might immediately proceed with implementation planning, establishing diagnostic networks, provider training programs, and ARIA monitoring protocols.

Central Italy faces intermediate challenges: adequate infrastructure exists but is concentrated in regional capitals, creating geographic access barriers for patients in peripheral areas. Central regions require modest capacity expansion and strengthened transport/telemedicine coordination to serve dispersed populations effectively.

Southern Italy faces severe constraints that require targeted policy interventions before meaningful implementation is possible. The current qualified center density (2–3 centers serving 15+ million inhabitants) makes the moderate scenario logistically infeasible without substantial concurrent infrastructure investment. This creates a critical equity choice: either (a) substantially invest in Southern Italian diagnostic network capacity before national reimbursement, ensuring equitable access; (b) implement nationally with foreknowledge that Southern patients will face extended waiting times and geographic barriers. However, this requires an explicit acknowledgment that the initial implementation will create geographic disparities, along with a robust commitment to eliminate them by 2028–2029.

The fundamental finding is that anti-amyloid therapy cannot be implemented equitably in Italy without targeted capacity investment in underserved regions. The moderate scenario's financial feasibility masks substantial infrastructure barriers that will overwhelm implementation efforts in Southern Italy without parallel investments in diagnostic centers, workforce training, and telemedicine infrastructure.

### 5.3 Policy recommendations

Based on this analysis, we might recommend the following integrated policy actions:

1. Adopt the Moderate Scenario as the official three-year reimbursement target (14,100 cumulative patients, €557.3 millions), with the expectation of expansion to 20,000–25,000 patients by 2030 as infrastructure capacity increases. This provides a realistic investment horizon for regional capacity planning.
2. Establish a National Anti-Amyloid Therapy Implementation Coordination Committee with representation from the SSN, regional health authorities, specialist societies, and patient advocacy groups. This committee should develop mandatory national diagnostic and monitoring guidelines, establish ARIA event reporting systems, and coordinate workforce training.
3. Allocate specific funding, estimated €40–50 million over three years, for Southern Italian capacity expansion, including: establishment of 8–12 new qualified CCDDs in high-population-density areas; training programs for neuropsychologists and specialized nurses; MRI and PET infrastructure investment; and telemedicine platform development for rural/remote patient monitoring.
4. Consider Caregiver and Patient Indirect Costs as Hidden Inequity. A critical gap is inadequate accounting for patient and caregiver indirect costs. The original budget model did NOT include transport, lodging, lost work productivity, or caregiver time costs, treating these as outside the healthcare system's financial responsibility. This represents a substantial equity blind spot, as these costs fall disproportionately on patients and families in underserved regions.
5. Project efficient and realistic Workforce Pipeline Planning. Moderate or Optimistic implementation requires deliberate workforce expansion across neurologists, geriatricians, neuroradiologists, and psychologists, with specific training timelines and geographic targeting. However, exact professional requirements depend on regional political decisions, economic sustainability, and quality targets—variables that two Italian consensus studies acknowledged but did not quantify [6,14]. Building this workforce network will benefit future disease-modifying therapies expected to reach the market within the next few years.
6. Take messages from International Experience. International models offer lessons: the United Kingdom's centralized approach created expertise but was hampered by geographic barriers [15]; Australia's decentralized strategy improved access but faced quality inconsistencies [16]; Israel achieved >80% biomarker concordance through standardized protocols and prospective equity monitoring from the outset of implementation [17,18]. Italy's greater regional fragmentation and existing geographic disparities suggest the Israeli model—with prospective equity infrastructure, standardized protocols, and documented outcome monitoring—is most applicable, though it would require more intensive central coordination and targeted investment in Southern Italy than Israel's centralized system did.
7. Implement mandatory management infrastructure and monitoring systems for adverse events and ARIA: 'Amyloid-Related Imaging Abnormalities (ARIA) represent a critical infrastructure burden. Symptomatic ARIA occurs in 2–5% of treated patients annually, with management costs ranging from €3000–€8000 per event, including hospitalization, neurological assessment, immunosuppression, and repeat imaging. Critically, ARIA surveillance requires access to high-quality brain MRI imaging and specialized neuroradiology interpretation, resources concentrated in Northern Italy. Southern regions lack adequate MRI capacity and neuroradiologists trained in ARIA recognition, creating a substantial equity barrier: patients in underserved regions may experience diagnostic delays in ARIA detection, potentially delaying life-saving interventions like immunosuppression or corticosteroids [8]
8. Establish blood-based biomarker approval processes as a priority, targeting p-tau181/p-tau217 approval by 2027. This will reduce diagnostic burden, expand the eligible population, and decrease diagnostic costs.
9. Develop integrated diagnostic networks linking non-specialist primary care settings with reference centers through telemedicine consultation models. This distributes the patient evaluation burden and improves geographic accessibility without requiring all diagnostic procedures to be performed at specialized centers.

These recommendations might ensure that adoption of effective disease-modifying therapies proceeds with explicit attention to financial sustainability, infrastructure realism, and health equity.

### 5.4 Limitations

This analysis has several important limitations. First, this is a non-empirical scenario analysis, not an outcomes impact study. Budget projections use deterministic modeling without probabilistic sensitivity analysis; real-world costs may vary ±15–25% due to drug pricing fluctuations, actual uptake rates, and variable ARIA incidence. However, the moderate scenario's cost (<1% of the SSN annual budget) provides a sufficient margin that a realistic variation is unlikely to alter scenario rankings. Second, our operationalized Proctor domain scores (0–2 scale) and success probabilities represent expert-judgment assessments of implementation readiness based on structural feasibility and organizational capacity. They are NOT statistically estimated probabilities derived from empirical data and should be interpreted as expert confidence judgments rather than predictive models. Third, the analysis excludes indirect costs (patient/caregiver transportation, lodging, lost productivity) and broader system benefits (reduced institutional care use, extended community dwelling), which would likely strengthen the economic case for implementation but also reveal additional equity barriers. Fourth, the CFIR equity assessment is qualitative and descriptive, not quantitative; barriers are identified through structured review rather than measured empirically. Fifth, the analysis assumes a stable Italian political and budgetary environment and continued SSN funding; a major economic recession or healthcare retrenchment could alter feasibility assessments. Finally, this analysis does not quantify real-world uptake rates or actual treatment efficacy in the Italian context; effectiveness estimates rely on published studies [3,4], which may not translate identically to diverse Italian populations with varying comorbidities and healthcare contexts

## 6 Conclusions

Anti-amyloid monoclonal antibodies are the first disease-modifying therapies demonstrating meaningful cognitive benefits in early Alzheimer's disease. Implementation is financially feasible under a moderate scenario (5–10–15% annual growth, 14,100 patients, €557.3 million), achieving high success probability while remaining affordable within SSN constraints.

However, geographic disparities in diagnostic infrastructure will inevitably favor Northern Italy unless targeted investment in underserved regions is prioritized. We recommend conditional adoption contingent on: (1) capacity building in Central and Southern regions; (2) phased regional rollout; (3) mandatory equity monitoring; and (4) commitment to eliminate geographic disparities by 2029.

These safeguards ensure anti-amyloid therapy advances Italy's position as an evidence-based healthcare system while addressing health equity—advancing the SSN's foundational commitment to equitable access based on clinical need rather than residence.

## Ethical considerations

No personal data were employed in this study.

## Funding statement

This work was funded by “10.13039/501100008530Fondo Regionale per l'Alzheimer e le demenze 2024–2026” according to D.G.R. 1424/2025.

## Declaration of generative AI and AI-assisted technologies

AI was not used in any phase of preparation of this paper including writing, figures, images and artwork.

## Data availability statement

This study is not associated with any data. All estimates are derived from published Italian epidemiological studies and national healthcare tariffs as cited in the manuscript.

## CRediT authorship contribution statement

**Maurizio Giorelli:** Writing – review & editing, Writing – original draft, Visualization, Validation, Supervision, Software, Resources, Methodology, Investigation, Formal analysis, Data curation, Conceptualization. **Pasquale Di Fazio:** Writing – review & editing, Conceptualization. **Tiziana Dimatteo:** Writing – review & editing, Conceptualization.

## Declaration of competing interest

The authors declare the following financial interests/personal relationships which may be considered as potential competing interests: Maurizio Giorelli reports financial support was provided by Council of Apulia Region. If there are other authors, they declare that they have no known competing financial interests or personal relationships that could have appeared to influence the work reported in this paper.
